# Involvement of HSP90 in ischemic postconditioning-induced cardioprotection by inhibition of the complement system, JNK and inflammation^1^

**DOI:** 10.1590/s0102-865020200010000005

**Published:** 2020-03-20

**Authors:** Dong-Xiao Wang, Zheng Huang, Qing-Jie Li, Guo-Qiang Zhong, Yan He, Wei-Qiang Huang, Xiao-Li Cao, Rong-Hui Tu, Jian-Jun Meng

**Affiliations:** I MD, Department of Cardiology, First Affiliated Hospital, Guang Xi Medical University, China. Manuscript preparation and writing.; II PhD, Department of Cardiology, First Affiliated Hospital, Guang Xi Medical University, China. Acquisition of data, manuscript preparation and writing.; III PhD, Department of Cardiology, Second Affiliated Hospital, Guang Xi Medical University, China. Analysis and interpretation of data, statistics analysis.; IV PhD, Department of Cardiology, First Affiliated Hospital, Guang Xi Medical University, China. Analysis and interpretation of data, technical procedures.; V PhD, Department of Geriatric Cardiology, First Affiliated Hospital, Guang Xi Medical University, China.; VI MD, Guang Xi Key Laboratory of Precision Medicine in Cardiocerebrovascular Diseases Control and Prevention, China. Acquisition of data, technical procedures.; VII PhD, Guang Xi Clinical Research Center for Cardiocerebrovascular Diseases, China. Acquisition of data, technical procedures.; VIII PhD, Department of Geriatric Cardiology, First Affiliated Hospital, Guang Xi Medical University, China. Conception and design of the study, final approval.; IX MD, Geriatric Health Care Center, First Affiliated Hospital, Guang Xi Medical University, China. Critical revision.

**Keywords:** HSP90 Heat-Shock Proteins, Ischemic Postconditioning, MAP Kinase Kinase 4, Inflammation, Rats

## Abstract

**Purpose:**

To investigate whether heat shock protein 90 (HSP90) is involved in complement regulation in ischemic postconditioning (IPC).

**Methods:**

The left coronary artery of rats underwent 30 min of occlusion, followed by 120 min of reperfusion and treatment with IPC via 3 cycles of 30s reperfusion and 30s occlusion. The rats were injected intraperitoneally with 1 mg/kg HSP90 inhibitor geldanamycin (GA) after anesthesia. Eighty rats were randomly divided into four groups: sham, ischemia-reperfusion (I/R), IPC and IPC + GA. Myocardial infarct size, apoptosis index and the expression of HSP90, C3, C5a, tumor necrosis factor (TNF)-alpha, interleukin (IL)-1β and c-Jun N-terminal kinase (JNK) were assessed.

**Results:**

Compared with the I/R injury, the IPC treatment significantly reduced infarct size, release of troponin T, creatine kinase-MB, and lactate dehydrogenase, and cardiomyocyte apoptosis. These beneficial effects were accompanied by a decrease in TNF-α, IL-1β, C3, C5a and JNK expression levels. However, all these effects were abrogated by administration of the HSP90 inhibitor GA.

**Conclusion:**

HSP90 exerts a profound effect on IPC cardioprotection, and may be linked to the inhibition of the complement system and JNK, ultimately attenuating I/R-induced myocardial injury and apoptosis.

## Introduction

Ischemic reperfusion injury caused by early reperfusion in the treatment of acute myocardial infarction often aggravates myocardial cell damage and further worsens the prognosis of patients^1,2^. Activation of the complement system is an important feature of ischemia-reperfusion (I/R) injury, and has received increasing attention^3,4^.

The complement system is composed of more than 30 plasma and cell membrane proteins and is designed to recognize hypoxia and ischemia ‘danger’ signals. In response to myocardial injury, the complement cascade is activated and initiates an inflammatory response involving the recruitment of polymorphonuclear leukocytes and the release of inflammatory cytokines. Indeed, the excessive activation of the complement and its products C3 and C5a play an important role in myocardial necrosis during myocardial I/R^5,6^. Moreover, the generation of the anaphylatoxins C3 and C5a further activates the c-Jun N-terminal kinase (JNK) signaling to aggravate inflammatory response^7^. Therefore, targeting the complement/JNK signaling may be a promising therapeutic strategy for I/R injuries.

Ischemic postconditioning (IPC) is defined as rapid, intermittent interruptions of blood flow in early reperfusion. Because of its timing, IPC could be used in clinical reperfusion settings involving direct angioplasty, and it has been shown to reduce infarct size and prevent reperfusion-induced endothelial dysfunction in humans^2,3^. Nowadays, IPC has been demonstrated to alleviate myocardial I/R injury in various models^8,9^. Several mechanisms have been implicated in this novel strategy, including the suppression of inflammation^10^. In fact, inhibition of complement-mediated inflammation is critical for ischemic preconditioning cardioprotection^11^. However, it has not been reported whether IPC has an inhibitory effect on the complement system.

Heat shock protein 90 (HSP90) plays a vital role in preconditioning cardioprotection^12^. Our laboratory recently showed that HSP90 is important for IPC-induced cardioprotection and that its activity may be linked to mitochondrial targeting of PKCepsilon^13^. Early studies have shown that the inhibition of HSP90 enhanced complement-mediated cell lysis^14,15^. Moreover, suppression of the JNK signaling pathway is critical for reducing infarct size in IPC^16^. However, it is unclear whether HSP90 is involved in the protective effects of IPC by suppressing the complement system and JNK signaling pathways on myocardial I/R injury.

Therefore, this study tested the hypothesis that IPC in rat attenuates myocardial I/R injury by inhibiting the complement system and JNK signaling via HSP90. The study examined the effects of IPC on infarct size, release of serum creatine kinase-MB (CK-MB), troponin T (cTnT) and lactate dehydrogenase (LDH) and cardiomyocyte apoptosis. The mRNA levels of the inflammatory cytokines including tumor necrosis factor (TNF)-α and interleukin (IL)-1β were examined by quantitative polymerase chain reaction (qPCR). HSP90, C3, C5a, JNK, TNF-α and IL-1β proteins levels were studied by Western blot analysis. The effects of the HSP90 inhibitor geldanamycin (GA) on IPC were also investigated.

## Methods

This study was conducted at the Experimental Animal Center of Guangxi Medical University. All experimental protocols used in this study were performed in accordance with the National Institutes of Health Guide for the Care and Use of Laboratory Animals and were approved by the Guangxi Medical University Animal Protection and Use Committee.

Eighty male Sprague Dawley rats weighing 250 ± 20 g were purchased from the Experimental Animal Center of Guangxi Medical University (Certificate No. SCXK (Gui) 2014-0002), China. The animals were maintained under standard laboratory conditions at 25 ± 2°C and a 12 hr light-12 hr dark cycle and were allowed unrestricted access to food and water.

### In vivo myocardial I/R model

The rats were anesthetized with sodium pentobarbital (50 mg/kg, intraperitoneally) and intubated. A rodent ventilator at a respiratory rate of 50 beats/min and 20 mL/kg body weight was used for positive pressure ventilation. The electrocardiogram (ECG) lead was then placed to monitor the Lead II electrocardiogram, the chest was opened in the left fifth intercostal space, and the 6-0 prolene suture needle was bypassed around the left anterior descending coronary artery (LAD) and passed through a small plastic tube to form a reversible LAD occlusion. Myocardial ischemia was induced by compressing the LAD by tightening the ligature around the plastic tube. After 30 min of ischemia, the ligature was dissected and allowed to reperfusion for 2 hr. The ECG monitored ST segment changes due to tightening or loosening of the ligation. At the end of reperfusion, the rats were euthanized and the anterior wall portion of the left ventricle near the apex and blood samples were obtained for further analysis.

### Experimental grouping

Eighty rats were randomly divided into the following 4 groups (n=20 in each group): (1) The sham group -the anterior descending artery was only threaded and not ligated); (2) the I/R group -the anterior descending branch was ligated for 30 minutes and then reperfused for 2 hours; (3) the IPC group -the anterior descending branch was ligated for 30 min, and then reperfused/ischemic for 30 seconds, 3 times and reperfused for 2 hours; and (4) the IPC + GA group – the rats received an intraperitoneal injection of GA (1 mg/kg) with the same operation as that for the IPC group.

### Measurement of myocardial infarct size

Separate experiments were performed to determine the infarction size (n = 5 for each group). At the end of the reperfusion, the LAD was retightened and 3% Evans blue dye was injected into the inferior vena cava to identify the area at risk. The stained heart was excised, frozen and cut into 2 mm slices, and incubated at 37 °C for 15 min in 1% 2,3,5-triphenyltetrazolium chloride (TTC, Sigma-Aldrich, US) to delineate the size of the infarction. Each slice was measured by digital imaging software (Image-Pro Plus version 6.0; Media Cybernetics, Bethesda, MD) to calculate the myocardial infarct size. The infarct size was expressed in terms of the infarct area/left ventricle (IFA/LV).

### Activities of CK-MB, LDH and cTnT in plasma

When the reperfusion was complete, 3 mL of right atrial blood was collected in a 5 mL vacuum tube and centrifuged at 2000g for 10 min. The supernatant was stored in liquid nitrogen. CK-MB, LDH and cTnT were assayed by an Automatic Analyser 7600 (Hitachi, Tokyo, Japan) using the following commercial kits: troponin T (cTn-T) ELISA Kit, L-lactate dehydrogenase (LDH) ELISA Kit and creatine kinase MB (CK-MB) Isoenzyme ELISA Kit (all acquired from CUSABIO, Shanghai, China).

### Terminal deoxynucleotidyl transferase dUTP nick end labelling (TUNEL) staining

A separate experiment was performed on four groups of five animals per group. In brief, paraformaldehyde-fixed heart tissue blocks were incubated with proteinase K, then washed, dehydrated, embedded in paraffin, and sectioned. Apoptotic cells were identified using the TUNEL assay kit (Roche Di-agnostics, Basel, Switzerland) according to the manufacturer’s protocol. Staining was observed using a microscope and at least five randomly selected fields of view were scored with apoptotic cells. The apoptotic cells were positive for TUNEL staining and showed brown or brownish yellow particles. The apoptosis index was expressed in terms of the TUNEL positive cells/total number of myocytes.

### Quantitation of mRNA expression

Total RNA from heart samples was extracted and purified using a Trizol reagent kit (Invitrogen, CA, US). Reverse transcription reactions were performed using a PrimeScript™ RT reagent Kit (Takara Bionic, Otsu, Japan). qPCR was performed on tumor necrosis factor (TNF)-α, interleukin (IL)-1β, using an SYBR standard qPCR mix (TaKaRa) on an ABI Prism 7500 Fast Real-Time System (Thermo Fisher Scientific, Shanghai, China). The reaction took place at 95°C for 30s, followed by 40 cycles of 95°C for 5s, and dissociation at 60°C for 30s. Glyceraldehyde-3-phosphate dehydrogenase (GAPDH) was used as the housekeeping genes. The mRNA levels were analyzed by the 2^-^ method. The following primers were used: TNF-α, sense primer: TGCCGAGTAGACCTCATAGTGACC, antisense primer: GCATGATCCGAGATGTGGAACTGG; IL-1β, sense primer: AGTGCTGCCTTGCTGTTCTTGAG, and antisense primer: ATCTCACAGCAGCATCTCGACAAG.

### Western blot analysis

Western blot was used to detect the protein expression of HSP90, JNK, C3 and C5a in the ischemic heart tissue. After successful establishment of the I/R model, the heart was removed, and then an appropriate amount of cardiac ischemic tissue was added to the RIPA tissue lysis buffer for homogenization. The protein concentration was determined by bicinchoninic acid assay (Beyotime, Shanghai, China) colorimetry. A 200 μg portion of each sample was subjected to gel electrophoresis. The protein was transferred to a nitrocellulose membrane and blocked with a 5% skim milk blocking solution in Tris- buffered saline Tween 20 (150 mM NaCl, 20 mM Trise HCl, 0.1% Tween 20, pH 7.4) for 3 hours at 25°C, and then incubated with the primary antibodies: anti-HSP90 (Abcam, Cambridge, UK), anti-JNK (Cell Signaling Technology, MA, US), anti-C3 (Abcam, Cambridge, UK), anti-C5a (Abcam, Cambridge, UK), anti-TNF-α (Cell Signaling Technology, MA, US), anti-IL-1β (Cell Signaling Technology, MA, US) and β-actin (Abcam, Cambridge, UK). After rinsing, it was incubated with horseradish peroxidase-labelled goat anti-rabbit immunoglobulin G and the chemiluminescent substrate and exposed to radiographic film. Image J 1.48 software was used to analyze the grey value of the protein bands. The relative expression level of the target protein was expressed by the grey value of the target protein/the grey value of the corresponding internal reference protein.

### Statistical analysis

All results were processed using SPSS 23.0 (SPSS, Inc, Chicago, IL, USA) statistical analysis software. Measurement data were expressed as mean ± standard deviation; multiple group means were compared using one-way analysis of variance, and the least significant difference method was used for comparison between groups. *P* < 0.05 was considered statistically significant.

## Results

Eighty rats were used in the present study. Due to the cardiogenic shock during the reperfusion and ventilator technical failure, three rats were excluded: One of them belonged to the IPC group and died unexplained, and the remaining two (one in the I/R group and one in the IPC + GA group) were excluded because of cardiogenic shock or tracheal obstruction. The presented results correspond to the remaining 77 rats.

### Expression of HSP90

**Figure 1:**
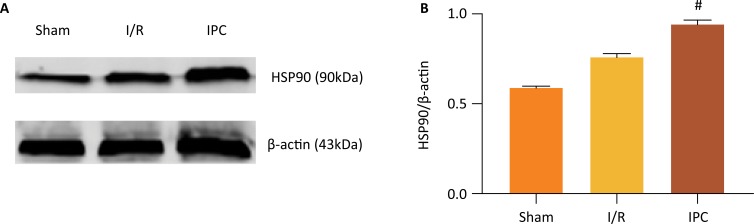
Effects of IPC on HSP90 protein expression. (A) Representative Western blots showing the expression of HSP90. (B) Relative expression of HSP90 protein. Values are presented as the mean ± standard deviation. #*P* < 0.05 vs. I/R group; n=5 for each group.

To determine whether HSP90 was involved in the postconditioning, we first examined the protein levels of HSP90 in the postconditioned hearts. As shown in Figure 1, IPC significantly increased the levels of the HSP90 protein compared with the I/R group (*P* < 0.05). Next, we discuss the effect of HSP90 on the cardioprotection of IPC by inhibiting HSP90 activity with the selective HSP90 inhibitor GA.

### Infarct size

**Figure 2:**
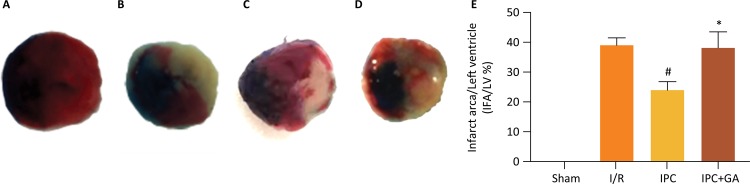
Effects of GA and IPC on myocardial infarct size after cardiac I/R injury (IFA/LV). (A) Sham group, (B) I/R group, (C) IPC group, (D) IPC+GA group, (E) Effects of GA and IPC on myocardial infarct size after cardiac I/R injury. GA = geldanamycin; I/R = ischemia - reperfusion; IPC = ischemic postconditioning; the results presented the mean ± standard deviation. #*P* < 0.05 vs. I/R group; *P < 0.05 vs. IPC group; n=5 for each group.

As shown in Figure 2, the infarct area was not found in the sham group. However, compared with I/R group (38.98 ± 2.53) %, the IPC group significantly reduced the IFA/LV ratio [(23.97 ± 2.82) %, *P* < 0.05]. The IFA/LV ratio (38.04 ± 5.58) % in the IPC + GA group increased significantly, compared with the IPC group (*P* < 0.05). Thus, GA counteracted the infarction- reducing effect of IPC.

### Myocardial injury

Serum enzymes such as CK-MB, LDH and cTnT are typical markers of myocardial injury. Their activities were significantly higher in the I/R group than in the sham group (Table 1). Compared with the I/R group, IPC significantly reduced the levels of CK-MB (0.75 ± 0.19 *vs*. 0.41 ± 0.10 ng/mL, respectively), LDH (23.04 ± 10.43 *vs*. 9.79 ± 0.50 mU/mL, respectively) and cTnT (171.22 ± 72.27 *vs*. 65.02 ± 11.42 pg/mL, respectively). The effect of the IPC was blocked by GA.

**Table 1:** Activities of CK-MB, cTnT and LDH in serum.

Groups	CK-MB (ng/mL)	cTnT (pg/mL)	LDH (mU/mL)
Sham	0.29 ± 0.05	23.00 ± 3.98	4.69 ± 2.62
I/R	0.75 ± 0.19	171.22 ± 72.27	23.04 ± 10.43
IPC	0.41 ± 0.10 ^#^	65.02 ± 11.42 ^#^	9.79 ± 0.50^#^
IPC+GA	0.63 ± 0.07 ^*^	146.11 ± 43.28 ^*^	20.87 ± 8.71^*^

GA = geldanamycin; I/R = ischemia - reperfusion; IPC = ischemic postconditioning; Data are presented as the mean ± standard deviation. ^#^ *P* < 0.05 *vs*. I/R group; ^*^ *P* < 0.05 *vs*. IPC group; n=5 for each group.

### Cardiomyocyte apoptosis

**Figure 3:**
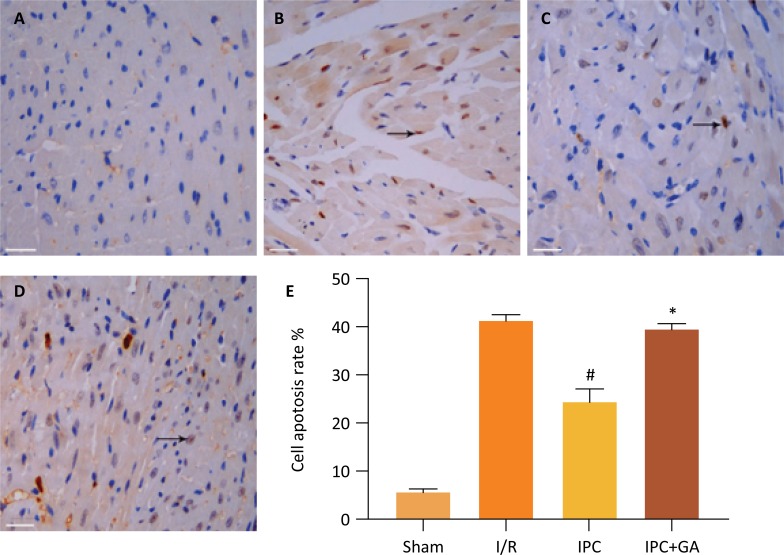
Effects of GA and IPC on apoptosis after myocardial I/R. (A) Sham group, (B) I/R group, (C) IPC group, (D) IPC+GA group. Apoptotic cardiomyocyte nuclei appear brown stained, whereas TUNEL-negative nuclei appear blue. Mean apoptotic index was counted in each group (A-D), results presented in a bar graph (E) are the mean ± standard deviation. Arrow indicates TUNEL-positive cells. TUNEL stain ×400, all bars = 20μm. Arrow indicates TUNEL - positive cells. #*P* < 0.05 *vs*. I/R group; *P < 0.05 *vs*. IPC group; n=5 for each group.

The apoptotic index of the cardiomyocytes was significantly lower in the IPC group (24.39 ± 2.81) % compared with the I/R group (41.46 ± 1.30) %, (*P* < 0.05, Fig. 3). There were no differences between the IPC+GA group (39.77 ± 1.26) % and the I/R group, suggesting that GA counteracted the apoptotic- and cardiomyocyte-limiting effects of IPC.

### Expression of inflammatory factors

**Figure 4:**
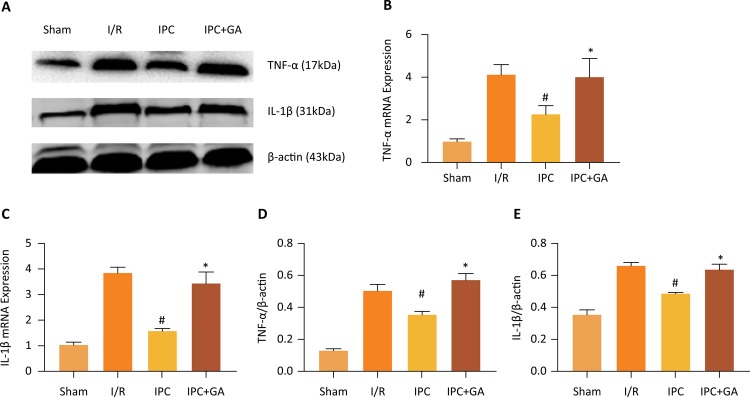
Effects of GA and IPC on TNF-α, IL-1β mRNA and protein expression. (A) Representative Western blots showing the expression of TNF-α and IL-1β. (B)(C) The mRNA levels of TNF-α and IL-1β were measured using qPCR in different groups. (D) Relative expression of TNF-α protein. (E) Relative expression of IL-1β protein. Values are presented as the mean ± standard deviation. #*P* < 0.05 *vs*. I/R group; *P < 0.05 *vs*. IPC group; n=5 for each group.

Both the mRNA and protein levels of IL-1β and TNF-α were detected. As shown in the Figure 4, the levels of IL-1β and TNF-α were significantly decreased in the IPC group compared with the I/R group (*P* < 0.05); the effects were restrained by GA. The results suggest that IPC protected the heart from I/R-related inflammatory response by suppressing the IL-1β and TNF-α levels. The application of GA before IPC eliminated the anti-inflammatory effect of IPC.

### Expression of C3, C5a and JNK

**Figure 5:**
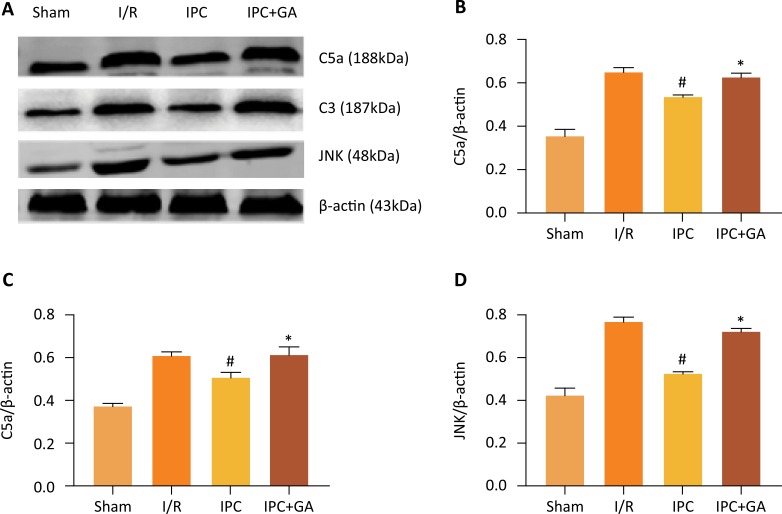
Effects of GA and IPC on C3, C5a and JNK protein expression. (A) Representative Western blots showing the expression of C3, C5a and JNK. (B) Relative expression of C5a protein. (C) Relative expression of C3 protein. (D) Relative expression of JNK protein. Values are presented as the mean ± standard deviation. #*P* < 0.05 *vs*. I/R group; *P < 0.05 *vs*. IPC group; n=5 for each group.

To determine whether the complement components-C3 and C5a and the JNK signaling pathway are modulated by IPC via HSP90, the protein expression levels of C3, C5a and JNK were examined. As shown in Figure 5, the activities of C3, C5a and JNK were simultaneously inhibited in the IPC group compared with the I/R group (50.75 ± 1.87 % *vs*. 61.49 ± 1.31 %, 53.24 ± 0.87 % *vs*. 65.33 ± 1.94 %, and 52.38 ± 0.63 % *vs*. 77.50 ± 1.67 %, respectively). The effects counteracted by GA, which showed IPC cardioprotection against I/R-induced inflammatory injury by restraining the complement/JNK signaling pathway, were in turn closely linked to HSP90.

## Discussion

In the present study, we explored the role and potential mechanisms of HSP90 and the complement system in IPC. We found that IPC significantly reduced the I/R-induced complement system and JNK activation by up-regulating the expression of HSP90, leading to further reductions in the myocardial infarction area, cardiomyocyte apoptosis, the release of cTnT, CK-MB and LDH, and the release of inflammatory mediators such as TNF-α and IL-1β, whereas, treatment with GA reversed the protection of IPC. Thus, it was proven for the first time that HSP90 is essential for inhibiting cardiac complement and immune inflammatory responses in IPC, possibly by inhibiting complement system activation and JNK. Our data reveal a novel mechanism of IPC cardioprotection.

Numerous studies have shown that the complement system is an important participant in the pathophysiological process of myocardial I/R injury^6,17,18^. Conversely, inhibiting complement system activation can significantly reduce myocardial I/R injury and myocardial infarct size^19-21^. In addition, ischemic preconditioning protects against myocardial I/R injury by inhibiting the complement system^11^. However, the inhibitory effect of IPC on the complement system is unclear. The experiments in the present study were designed to determine the interaction between IPC and the complement system. We found that the levels of C3 and C5a were significantly reduced by IPC, indicating that the inhibiting complement induced IPC cardioprotection.

At present, the activation of the complement system and the JNK signaling pathway and subsequent inflammatory responses in myocardial I/R injury are highly valued. Once activated, the released C3 and C5a, can recruit concentrated granulocytes and monocytes to the injured myocardia through chemotaxis, aggravating the inflammatory response^5,22^. Moreover, the C3 and C5a can activate the JNK signaling pathways to promote the expression of downstream TNF-α and IL-1β 7. As risk signals for acute inflammation following myocardial infarction, these chemotactic factors trigger cardiomyocyte apoptosis and other I/R injuries^23,24^. Therefore, the JNK signaling is an important pathway for regulating inflammation and apoptosis^25,26^. Thus, in this study, we verified that the anti-inflammatory and cardioprotective effects of IPC might be attributed to the suppression of the JNK pathway. Nevertheless, the underlying mechanisms still need to be elucidated.

As a molecular chaperone, the main function of HSP90 is to regulate the stability and activity of the client protein, which is pivotal for maintaining cell structure and function and inhibiting apoptosis^27,28^. Recent studies have shown that HSP90 binds to the chaperone-mortalin protein to attenuate complement-mediated cell lysis^29^, while HSP90 inhibitors promote this process^14,15^. Therefore, HSP90 is an emerging therapeutic target in I/R injury and cardioprotection^12,13^. Moreover, our laboratory recently reported that the HSP90-mediated mitochondrial import of PKCepsilon played a central role in the IPC-induced cardioprotection from I/R injury^13^. Although increasing evidence suggests that HSP90 is linked to IPC, no study has addressed the interaction between HSP90 and the complement system as well as the JNK signaling in IPC. In the present study, we tested whether postconditioning-inhibited cardiac inflammation was mediated by the complement system and the JNK signaling via HSP90. We found that the levels of C3, C5a, JNK and inflammatory factors such as TNF-α and IL-1β were relatively low in the IPC group, but they were significantly higher in the IPC + GA group, indicating that HSP90 is important for the inhibition of the complement system and JNK during IPC. To the best of our knowledge, this study is the first description of the correlation between HSP90, and the complement system and the JNK signaling in IPC, suggesting a possible anti-inflammation mechanism for IPC cardioprotection. However, more experiments are required to elucidate the detailed mechanism underlying this protection.

## Conclusions

HSP90 exerts a profound effect on IPC cardioprotection, and its function may be linked to the inhibition of complement system, JNK and inflammatory responses, ultimately attenuating I/R-induced myocardial injury and apoptosis. Our observations complement the literature on IPC-related cardiac protection against I/R
